# Safety and efficacy of transseptal puncture guided by real-time fusion of echocardiography and fluoroscopy

**DOI:** 10.1007/s12471-016-0937-0

**Published:** 2016-12-13

**Authors:** S. Afzal, V. Veulemans, J. Balzer, T. Rassaf, K. Hellhammer, A. Polzin, M. Kelm, T. Zeus

**Affiliations:** 1 Division of Cardiology, Pneumology, and Vascular Medicine, Department of Medicine, University Hospital Düsseldorf, Düsseldorf, Germany; 2 Department of Cardiology, University Hospital Essen, Westgerman Heart-and Vascular Centre, Essen, Germany

**Keywords:** EchoNavigator system Release II, MitraClip implantation, Left atrial appendage closure, Transseptal puncture

## Abstract

**Aims:**

Visual guidance through echocardiography and fluoroscopy is crucial for a successful transseptal puncture (TSP) in a prespecified region of the fossa ovalis. The novel EchoNavigator system Release II (EchoNav II, Philips Healthcare, Andover, Massachusetts, USA) enables the real-time fusion of fluoroscopic and echocardiographic images. We evaluated this new imaging method in respect to safety and efficacy of TSP during MitraClip implantation and left atrial appendage closure.

**Methods:**

Forty-four patients before (−EchoNav) and 44 patients after (+EchoNav) the introduction of real-time fusion were included in our retrospective, single-centre study. The primary endpoint was the occurrence of adverse events due to TSP. Secondary endpoints were successful puncture at the prespecified region and time until TSP (min).

**Results:**

In both groups TSP was performed successfully in the prespecified region and no adverse events occurred during or due to the accomplishment of TSP. Time until TSP was significantly reduced in the +EchoNav group in comparison with the EchoNav group (18.48 ± 5.62 min vs. 23.20 ± 9.61 min, *p* = 0.006).

**Conclusions:**

Real-time fusion of echocardiography and fluoroscopy proved to be as safe and successful as standard best practice for TSP. Moreover, efficacy was improved through significant reduction of time until TSP.

## Introduction

**Fig. 1:**
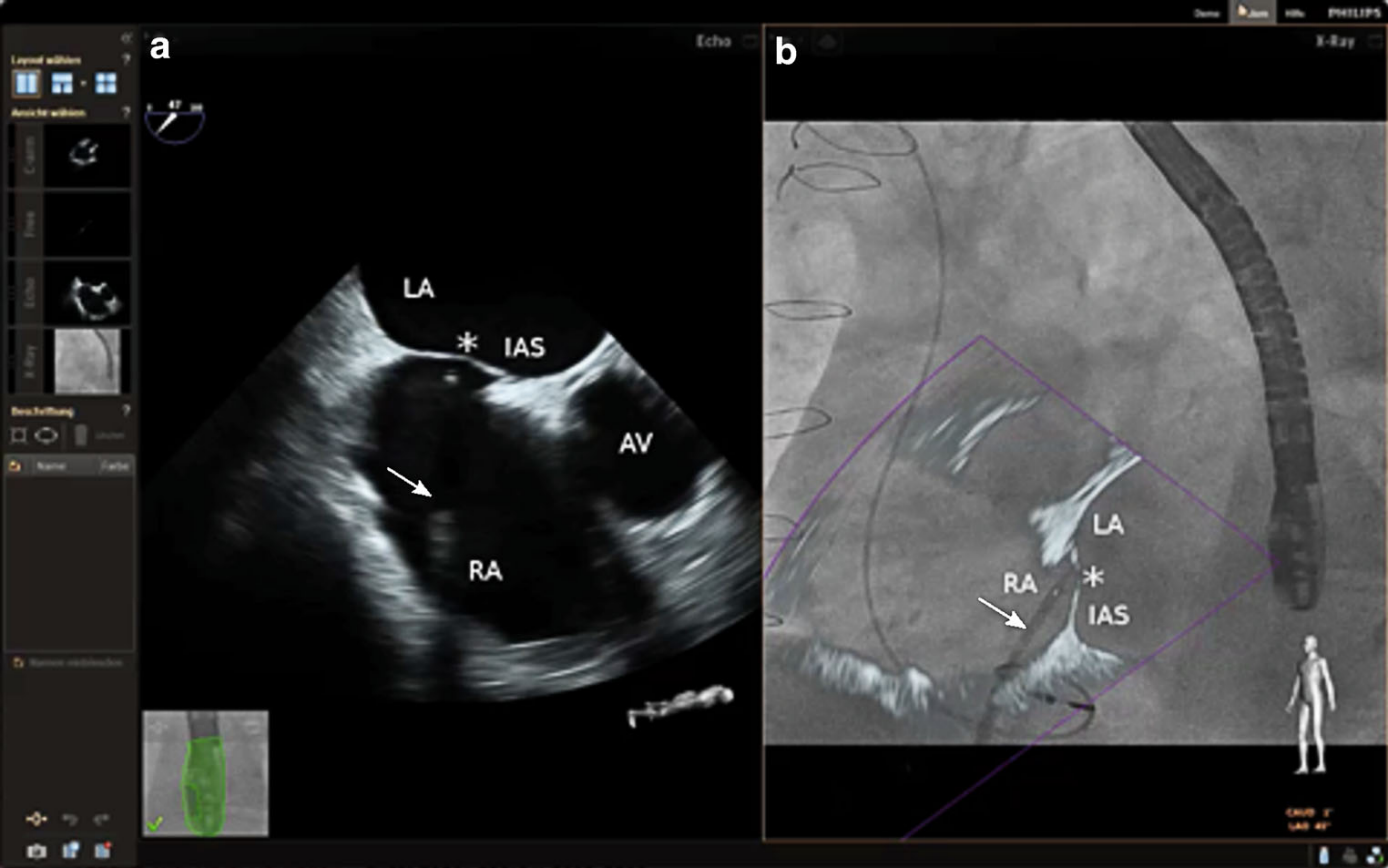
Transseptal puncture is demonstrated during LAA closure guided with EchoNav II. Transoesophageal echocardiogram in short-axis view shows tenting (*) of the interatrial septum on the left side (**a**). EchoNavigator system Release II allows real-time fusion of echocardiographic and fluoroscopic images (**b**). *AV* aortic valve, *IAS* intra-atrial septum, *LA* left atrium, *RA* right atrium; Arrows are pointing at the TSP sheath

**Fig. 2:**
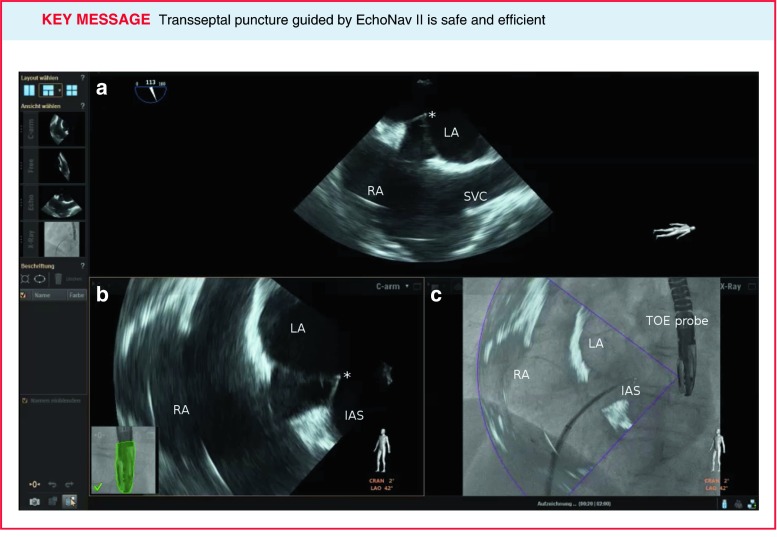
Transseptal puncture is depicted during LAA closure guided with EchoNav II. Transoesophageal echocardiogram in bicaval view shows tenting (*) of the interatrial septum (**a**); **b** reflects the calibration with the TEE probe and the 2D TEE image in the same anatomical direction as the C arm; **c** displays real-time fusion of echocardiographic and fluoroscopic images. *AV* aortic valve, *IAS* intra-atrial septum, *LA* left atrium, *RA* right atrium, *SVC* superior vena cava

Percutaneous interventions such as MitraClip (Abbott Vascular, Santa Clara, California, USA) implantation and left atrial appendage (LAA) closure require a precise transseptal puncture (TSP) in a prespecified region of the interatrial septum [1, 2]. Therefore, visual guidance through 2D and/or 3D echocardiography in addition to fluoroscopy is essential for a successful performance, since only echocardiography depicts anatomic soft tissue structures [3]. Until now, transoesophageal echocardiography (TEE) images/measurements and fluoroscopy are displayed on separate monitors and the interventionalist has to merge this information mentally. This mental coordination of both modalities is a challenging task [4, 5]. A novel imaging technology combines real-time echocardiographic and fluoroscopic images (EchoNavigator system Release II (EchoNav II), Philips Healthcare, Andover, Massachusetts, USA), presenting both in an interactive view (Figs. 1 and 2). To date this imaging technique in adult patients has been described in case reports only [6, 7]. In this observational study, we report data regarding the safety and efficacy of performing TSP with the guidance of real-time fused echocardiographic and fluoroscopic images for the first time.

## Materials and methods

### Real-time fusion imaging

The recently introduced EchoNav II allows the display of fluoroscopic and echocardiographic images in real-time overlay. For a successful real-time fusion, the echocardiography probe needs to be localised and tracked in respect to the fluoroscopy C‑arm angulation which is achieved with a calibration algorithm. After successful co-registration, the movements of the C‑arm and the TEE probe are detected, and real-time overlay of echocardiography and fluoroscopy is updated. Additional markers can be set for better visual guidance [8]. These markers serve as orientation. They are not combined with automatic tissue tracking and therefore remain static within the fluoroscopic picture. Compared with these static markers, the real-time fusion imaging gives continuously exact echo data within the fluoroscopic image allowing precise tracking of the soft tissue anatomy during breathing and heart movement on the fluoroscopic image.

### Study design

**Fig. 3:**
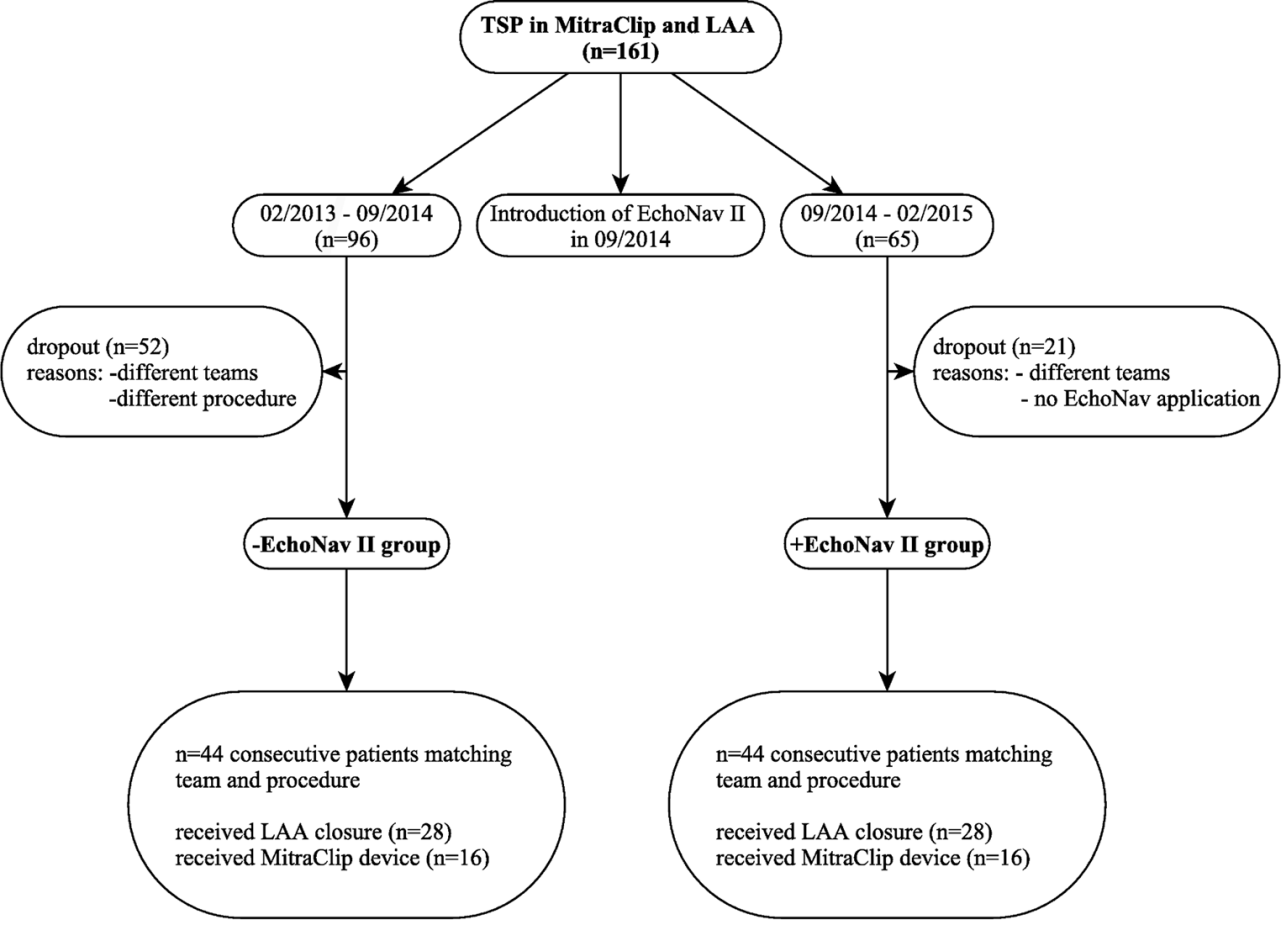
Consort diagram gives an overview of allocated patients for both, +EchoNav II group (with application of EchoNav II during MitraClip or LAA closure procedures) and -EchoNav II group (without application of EchoNav II during MitraClip or LAA closure procedures). *LAA* left atrial appendage, *EchoNav II* Echonavigator system Release II

Real-time fusion was introduced in our clinic in September 2014. From September 2014 until February 2015 65 patients received either a MitraClip or a LAA occluder (Amplatzer Cardiac Plug, St Jude Medical, St. Paul, Minnesota, USA) implantation. A total of 44 interventions were performed with the guidance of real-time fused echocardiographic and fluoroscopic images and by a fixed team of always the same interventionalist and experienced echocardiographer (+EchoNav group). The second structural team in our institution is mainly working without echonavigation, therefore follows a different strategy and is less experienced with echonavigation. From February 2013 to September 2014, 44 consecutive patients matching the type of procedure and the team members were defined as the control group (−EchoNav group). Therefore, a total of 88 patients were included in the observational single-centre analysis; 16 patients out of each group received a MitraClip implantation and 28 patients LAA closure (Fig. 3). Interventions started with arterial and venous access according to local standard operating procedures for MitraClip and LAA closure. After inserting the TEE probe, calibration of fluoroscopy and TEE probe was performed during standard rotation of the C‑arm for the TSP sheath placement and orientation in the superior vena cava. Calibration was achieved within a few seconds (<10 s). Afterwards TSP was performed in LAO 45° C‑arm angulation with a real-time overlay of 110° TEE image of the left and right atrium and the atrial septum. While Fig. 1 shows TSP in the short-axis view, Fig. 2 demonstrates the bicaval view during TSP. Both views were used for image registration and fusion. TSP was aimed inferiorly and posteriorly for LAA closure and at a height of 4.0–5.0 cm above the mitral valve annulus plane for MitraClip. The location of septal puncture was re-evaluated directly after sheath placement. Success was thereby defined as inferior and posterior sheath placement within the fossa ovalis for LAA closure and as sheath height of 4–5 cm above the mitral valve annulus for the MitraClip procedure.

The primary endpoint was considered to be the occurrence of adverse events during the performance of TSP. Adverse events were defined as pericardial effusion, stroke, air embolism, arrhythmia and death. Secondary endpoints were successful puncture at the prespecified region and time until TSP (min). Time until TSP comprised the interval from femoral vascular access in local anaesthesia until successful puncture of the septum in the prespecified region. The retrospective analysis was approved by the ethics committee of the Heinrich Heine University Düsseldorf (NCT02608008).

### Statistical analysis

The analysis was performed with SPSS for Windows PASW statistic, version 20.0.0.0 (SPSS Inc., Chicago, Illinois)*. *Datasets were statistically analysed by the independent samples Student’s t‑test. For categorical baseline characteristics of the patients and procedural outcome the chi-square test was calculated. Continuous data were expressed as mean and standard deviation. *P*-values below 0.05 were assumed to be significant.

## Results

### Patient characteristics

The baseline characteristics of the patients are depicted in Table 1. There were no significant differences between both groups.

**Table 1:** Baseline characteristics of the patients

	MitraClip/LAA-Oc (+EchoNav) *n* = 44	MitraClip/LAA-Oc (−EchoNav) *n* = 44	*p*-value*
Male gender, *n* (%)	25 (56.81%)	26 (59.09%)	0.83
Age, years ± SD	77 ± 7.22	75.77 ± 8.11	0.182**
Height, cm ± SD	169 ± 9.30	170 ± 8.78	0.526**
Weight, kg ± SD	78.31 ± 12.13	78.86 ± 15.13	0.852**
Chronic heart failure, *n* (%)	28 (63.63%)	25 (56.81%)	0.51
Chronic kidney disease, *n* (%)	29 (65.90%)	22 (50%)	0.13
Atrial fibrillation, *n* (%)	42 (95.45%)	37 (84.09%)	0.08
Coronary artery disease, *n* (%)	31 (70.45%)	33 (75%)	0.63
Prior myocardial infarction, *n* (%)	31 (70.45%)	28 (63.63%)	0.50
Previous CABG, *n* (%)	9 (20.45%)	10 (22.72%)	0.80
Previous PCI, *n* (%)	28 (63.63%)	25 (56.81%)	0.51
Hypercholesterolaemia, *n* (%)	23 (52.27%)	26 (59.09%)	0.60
Hypertension, *n* (%)	40 (90.90%)	41 (93.18%)	0.69
Diabetes mellitus, *n* (%)	12 (27.27%)	14 (31.81%)	0.64
COPD, *n* (%)	8 (18.18%)	9 (20.45%)	0.79
Aspirin, *n* (%)	17 (38.63%)	27 (61.36%)	0.86
P2 Y12 inhibitor, *n* (%)	18 (40.91%)	14 (31.81)	0.38
OAC/NOAC, *n* (%)	27 (61.36%)	19 (43.18%)	0.09
DAPT, *n* (%)	8 (18.18%)	3 (6.82%)	0.11
Triple therapy, *n* (%)	5 (11.36%)	3 (6.82%)	0.46

*SD* standard deviation, *COPD* chronic obstructive pulmonary disease, *PCI* percutaneous coronary intervention, *CABG* coronary artery bypass graft, *OAC* oral anticoagulants, *NOAC* novel oral anticoagulants, *DAPT* dual antiplatelet therapy

*Pearson’s chi-squared test

**independent samples Student’s t‑test

### Primary endpoints

No adverse events occurred during or due to TSP in both groups (Table 2). Two adverse events occurred after transseptal puncture: An LAA-Occluder device was dislocated and successfully retrieved from the left atrium with a standard snare in the +EchoNav group, and an intermittent third-degree AV block occurred periprocedurally during LAA closure in the −EchoNav group.

**Table 2:** Complications due to transeptal puncture in the +EchoNav and −EchoNav group

	MitraClip/LAA-Oc (+EchoNav) *n* = 44	MitraClip/LAA-Oc (−EchoNav) *n* = 44
Successful TSP, *n* (%)	44 (100%)	44 (100%)
Air embolism, *n*	0	0
Pericardial effusion, *n*	0	0
Arrhythmia, *n*	0	0
Stroke, *n*	0	0
Death, *n*	0	0

*TSP* transseptal puncture

### Secondary endpoints

**Fig. 4:**
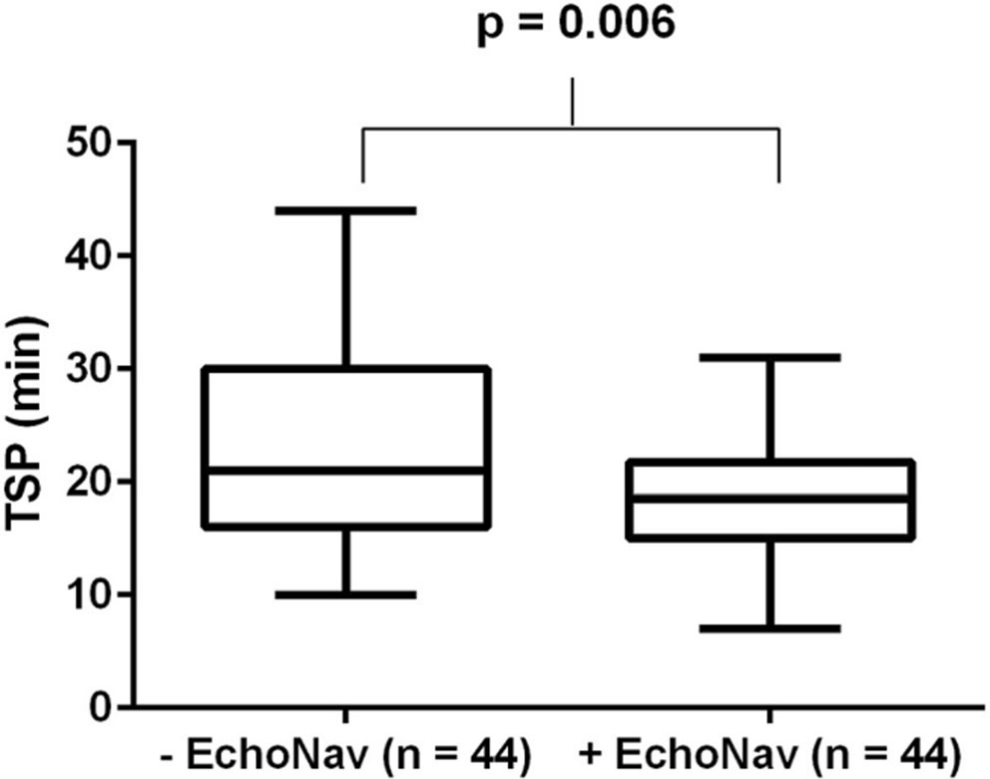
The distribution of time until TSP is shown as a box plot with and without the use of the EchoNavigator System Release II. The bars represent the whiskers from the minimum to the maximum of all data of the respective group. The interquartile range (IQR) is 6.75 for the +EchoNav and 14 for the −EchoNav group. The median was determined as 18.5 min for the +EchoNav and 21.0 min for the −EchoNav group. Time until transseptal puncture (TSP) was significantly reduced in the +EchoNav group compared with the −EchoNav group (*p = 0.006 for an independent-samples t‑test)

All TSP were performed successfully in both groups (success rate 100% in +EchoNav/−EchoNav group). Fig. 4 shows the distribution of time until TSP as a box plot with and without the use of the EchoNavigator System Release II. Time until successful TSP was significantly decreased in the +EchoNav group in comparison with the −EchoNav group (18.48 ± 5.62 min vs. 23.20 ± 9.61 min, *p* = 0.006).

## Discussion

Fusion of different imaging modalities is a novel approach to facilitate interventions in structural heart diseases. The first technical attempts were able to show markers on fluoroscopic images in real-time, which have been placed within echocardiographic images (EchoNav I, Echonavigator System Release I, Philips Healthcare, Andover, Massachusetts, USA) [9]. A previously published study reported benefits using EchoNav I during MitraClip implantation. Although the radiation dose was almost similar in the +/−EchoNav group, a trend in minimising procedural time could be observed [10]. Another recent study showed that EchoNav I guidance reduces the radiation dose and the fluoroscopy time during LAA closure without increasing the overall procedure time and the rate of periprocedural complications [11].

Advancement in technology leads to EchoNav II. In addition to markers, this tool simultaneously presents the echocardiographic image as overlay on the fluoroscopic images. Until now, only case reports have been published which highlight the precision and features of real-time fusion of echocardiographic and fluoroscopic images. One report depicted possible advantages of using EchoNav II during TSP in general [7]. Another case report presented the successful application of EchoNav II during the complex procedure of transapical mitral valve-in-valve implantation of a Sapien III prosthesis (Edwards Lifesciences, Irvine, California, USA) [6]. Furthermore, the use of EchoNav II has been described as feasible and safe in paediatric patients with congenital heart diseases such as atrial sepal defect [12]. Hereby, the use of fusion imaging reduced radiation dose and fluoroscopy time whereas the overall procedural time remained the same.

However, no systematically analysed data have been reported so far in adults. Therefore we aimed to investigate the application of EchoNav II guidance of TSP during MitraClip procedure and LAA closure in a relevant cohort of patients and on the performance of TSP as a pivotal periprocedural step. Our retrospective analysis of 88 patients revealed the following: No adverse events during or due to TSP in the +EchoNav and in the −EchoNav group, thus showing safety of the technology, a success rate of 100% of TSP within the prespecified region in the +EchoNav and in the −EchoNav group and a significant reduction of time until successful TSP in the +EchoNav group compared with the −EchoNav group, thus showing efficacy.

Two adverse events occurred after and independently from TSP during LAA closure. LAA-Occluder dislocation occurred in the +EchoNav group and an intermittent third-degree AV-block occurred periprocedurally during LAA closure in the −EchoNav group. Concerning occurrence and characteristics of adverse events they are consistent with results from earlier studies [13, 14]. Time to TSP of around 20 min seems to be quite long at first sight, but as is stated in the Methods section, we started to count from the exact beginning of the procedure with local anaesthesia. In summary, the application of real-time fused echocardiographic and fluoroscopic images for the guidance of TSP can be regarded as safe and efficient.

Looking into the future of fusion imaging we believe that the current version could be improved by displaying markers, which move with systole and diastole and breathing motion (tissue tracking). Moreover, automatic formation of 3D heart models with real-time overlay could improve orientation. The next developmental steps could also be automatic measurement algorithms (LAA annulus and landing zone, aortic annulus, sinus of Valsalva etc.). All this could increase relevance and benefit of this technical development.

## Limitations

We determined TSP as a crucial step in several structural heart interventions. As it is a standardised manoeuver, duration of TSP, success of TSP and adverse events can be analysed precisely. Since our study focused only on LAA closure and MitraClip procedures with the inclusion of 88 patients in a retrospective fashion, it is underpowered concerning safety. However, our results allow a robust statement concerning efficacy and give a strong indication concerning safety. Larger randomised trials are needed to confirm overall benefits for the MitraClip procedure as well as for LAA closure using EchoNav II. Moreover its value for other structural interventions with TEE guidance should be analysed in the future.

Since we conducted a retrospective cohort study, learning curve has to be discussed. However, the highly experienced team members (echocardiographer and interventionalist) had conducted several hundred TSP procedures before initiation of Echonav II and therefore have reached the flat part of their learning curve concerning TSP. The combination of echocardiographic information within fluoroscopic images was introduced with EchoNav release I in 2012. Only static markers could be fused with real-time fluoroscopic images by then. However, the team was familiar with imaging characteristics and technical aspects such as calibration of fluoroscopic and echocardiographic images in 2014. Therefore, no relevant learning curve has to be taken into consideration when Release II was introduced. Thus we concluded that the significant reduction in time until TSP could be attributed to innovation in fusion technology.

## Conclusion

Guidance of TSP with real-time fusion during the MitraClip procedure and LAA closure proved to be safe and efficient in terms of reduction of time until TSP. Larger randomised trials are needed to confirm overall benefits for MitraClip procedure as well as for LAA closure using real-time fusion.
